# Genetic evaluation and pregnancy outcome of fetuses with digestive system malformations: an eight-year single-center retrospective study

**DOI:** 10.3389/fped.2026.1798935

**Published:** 2026-05-08

**Authors:** Jianlong Zhuang, Nan Huang, Wenli Chen, Yu'e Chen

**Affiliations:** 1 Prenatal Diagnosis Center, Quanzhou Women's and Children's Hospital, Quanzhou, China; 2 The Teaching and Research Office of Clinical Laboratory Medicine, Quanzhou Medical College, Quanzhou, China; 3 Department of Ultrasound, Quanzhou Women's and Children's Hospital, Quanzhou, China

**Keywords:** chromosomal microarray analysis, copy number variants, digestive system malformations, karyotype analysis, prenatal diagnosis

## Abstract

**Background:**

Limited reports have investigated the genetic etiology of fetuses with digestive system malformations (DSMs). Our initial aim was to describe supplement data of fetal DSMs and further elucidate the genotype-phenotype correlations in fetuses with DSMs.

**Methods:**

A total of 7,497 pregnancies with various high-risk factors undergoing prenatal diagnosis were enrolled at Quanzhou Women's and Children's Hospital from 2017 to 2024. Among these, 76 fetuses diagnosed with DSMs via prenatal ultrasound were collected for further analysis. All subjects underwent amniocentesis, followed by karyotype analysis and chromosomal microarray analysis (CMA).

**Results:**

Karyotype analysis identified 3 cases of trisomy-21, 1 case of trisomy-18, and 1 case of balanced translocation t(10;11), reaching a chromosomal aberration detection rate of 6.58% (5/76). The chromosomal aneuploies detected by karyotype were confirmed by CMA. Additionally, CMA identified 6 cases of likely pathogenic/pathogenic CNVs (pCNVs/lpCNVs) that were missed by karyotype analysis, including 17q12 microdeletion/microduplication, 16p11.2 microduplication, 22q11.21q11.22 microdeletion, 2q13 microdeletion, and 16p13.11 microduplication, yielding an incremental diagnostic rate of 7.89% (6/76) for CMA over karyotype analysis (*P* = 0.031).

**Conclusion:**

Several pCNVs/lpCNVs that associate with DSMs were identified. Our findings may strengthen the association between 16p11.2 microduplication syndrome and DSMs, and the correlation between 16p13.11 microduplication syndrome and intestinal malrotation.

## Introduction

Fetal digestive system malformations (DSMs) are prevalent congenital structural anomalies, with an annual incidence of approximately 15 per 10,000 live births in Europe (1). Prenatal ultrasound can effectively detect and classify DSMs, which include omphalocele, esophageal atresia, Hirschsprung disease, intestinal atresia, gastroschisis, and anorectal malformations (2). Most affected fetuses develop postnatal symptoms such as feeding difficulties, delayed defecation, and vomiting, which may be life-threatening in severe cases requiring surgical intervention (1, 2). The genetic basis of DSMs includes chromosomal abnormalities, copy number variants (CNVs), and syndromic associations, among which chromosomal abnormalities are a major contributing factor to DSMs, with frequent chromosomal anomalies including trisomies 21, 18, 13, and Turner syndrome (3–5). Chromosomal microarray analysis (CMA) offers significant advantages in rapidly and accurately detecting CNVs, regions of homozygosity (ROH), uniparental disomy (UPD), and low proportion mosaicism, and has been recommended as a first-line tool for prenatal diagnosis in pregnant women undergoing amniocentesis (6–8). A previous study indicated that CMA yields an approximately 3.17% higher detection rate of pCNVs compared with karyotype analysis (9). Several CNVs potentially associated with DSMs were described, such as 17q12 microdeletion, 4q22.3 microdeletion, 8p23 microdeletion, and 12q23.1 microduplication (3, 10, 11). However, data on the application of CMA for CNVs screening in fetuses with DSMs remain limited, as well as the subsequent follow-up of pregnancy outcomes.

In this study, 76 fetuses with DSMs among the 7,497 pregnancies from Chinese population were enrolled. This study aims to provide additional genetic information and pregnancy outcomes of fetuses with DSMs and gain more insight into the genotype and phenotype relationship of fetuses with DSMs.

## Materials and methods

### Subjects

A total of 7,497 pregnancies undergoing prenatal diagnosis at Quanzhou Women's and Children's Hospital due to various high-risk factors were enrolled from April 2017 to December 2024. Inclusion criteria were singleton pregnancies with fetal DSMs detected by ultrasound. Exclusion criteria included multiple pregnancies and digestive system abnormalities presenting as soft markers (e.g., increased intestinal echogenicity). All 76 enrolled subjects provided written informed consent prior to undergoing karyotype analysis and CMA. Cases were divided into three subgroups (9): isolated DSMs (*n* = 33), DSMs combined with other ultrasound-detected structural anomalies (*n* = 9), and 34 cases with DSMs combined with ultrasound soft marker anomalies (*n* = 34). The study was approved by the Institutional Ethics Committee of Quanzhou Women's and Children's Hospital (approval number: 2024No.100).

### Karyotype analysis

Approximately 30 mL of amniotic fluid was collected from each fetus, with 20 mL inoculated into amniotic fluid culture medium and cultured according to standard operating procedures. Cultured amniotic fluid cells were harvested using the Sinochrome Chromprep II automatic chromosome harvesting system (Shanghai Lechen Biotechnology Co., Ltd.) following the previous standard protocols (12). Conventional G-banded karyotyping was performed at a 400-band resolution. For each case, 30 karyotypes were counted and 5 were analyzed; for cases suspicious for mosaicism, no less than 50 metaphases were analyzed. Chromosome karyotype analysis and nomenclature were performed in accordance with the International System for Human Cytogenomic Nomenclature (ISCN 2020) (13).

### Genomic DNA extraction

Approximately 10 mL of amniotic fluid was collected from each fetus. Peripheral blood samples from parents were collected if additional CMA analysis was required. Genomic DNA was extracted using the QIAamp DNA Blood Kit (QIAGEN, Germany) according to the manufacturer's instructions (www.qiagen.com).

### Chromosomal microarray analysis

CMA was performed using the single-nucleotide polymorphism (SNP)-based Affymetrix Cytoscan 750K chip (Life Technologies, USA) as described in our previous study (14). Briefly, 250 ng of genomic DNA was digested with NspI enzyme, end-repaired with ligase, and amplified by polymerase chain reaction (PCR). The amplified products were fragmented, labeled with biotin, and hybridized to the chip. Hybridized chips were washed, stained, and scanned for fluorescence signals. SNPs and CNVs were analyzed using Genotyping Console and Chromosome Analysis Suite software. In routine clinical practice, only CNVs involving deletions ≥200 kb and duplications ≥500 kb are reported. CNV pathogenicity was interpreted using databases including the Database of Genomic Variants (DGV, http://dgv.tcag.ca/dgv), Online Mendelian Inheritance in Man (OMIM, https://omim.org/), DECIPHER (https://decipher.sanger.ac.uk/), PubMed (https://www.ncbi.nlm.nih.gov/pubmed/), and local databases. CNVs were scored and classified as pathogenic (P, ≥0.99), likely pathogenic (LP, 0.90 to 0.98), variant of uncertain clinical significance (VUS, −0.89 to 0.89), likely benign (−0.90 to −0.98), or benign (≤−0.99) in accordance with the joint consensus guidelines of the American College of Medical Genetics and Genomics (ACMG) and the Clinical Genome Resource (ClinGen) (15).

### Statistical analysis

Data analysis was performed using SPSS 20.0 software. Intergroup comparisons were conducted using the chi-square test or Fisher's exact test. A *P*-value < 0.05 was considered statistically significant.

## Results

### Subjects' information

A total of 76 pregnancies with fetal DSMs were enrolled, corresponding to an incidence of 1.01% (76/7,497). All subjects successfully underwent amniocentesis and were categorized into three groups: isolated DSMs (*n* = 33), DSMs combined with other structural anomalies (*n* = 9), and DSMs combined with soft marker anomalies (*n* = 34). Furthermore, the included cases were further divided into five groups according to anatomical location: gastric and duodenal anomalies (*n* = 27), small intestinal anomalies (*n* = 19), hepatobiliary anomalies (*n* = 15), abdominal wall anomalies (*n* = 8), and other subgroups (*n* = 7).

### Karyotype analysis results

Karyotype analysis and CMA were successfully completed for all 76 cases. Karyotype analysis identified 4 cases of chromosomal aneuploies (3 cases of T-21 and 1 case of T-18) and 1 case of balanced translocation between chromosomes 10 and 11 [46,XN,t(10;11)(q26.3;q13.1)], which was not detected by CMA. The overall chromosomal aberration detection rate by karyotype analysis was 6.58% (5/76).

### Chromosomal microarray analysis results

**Figure 1:**
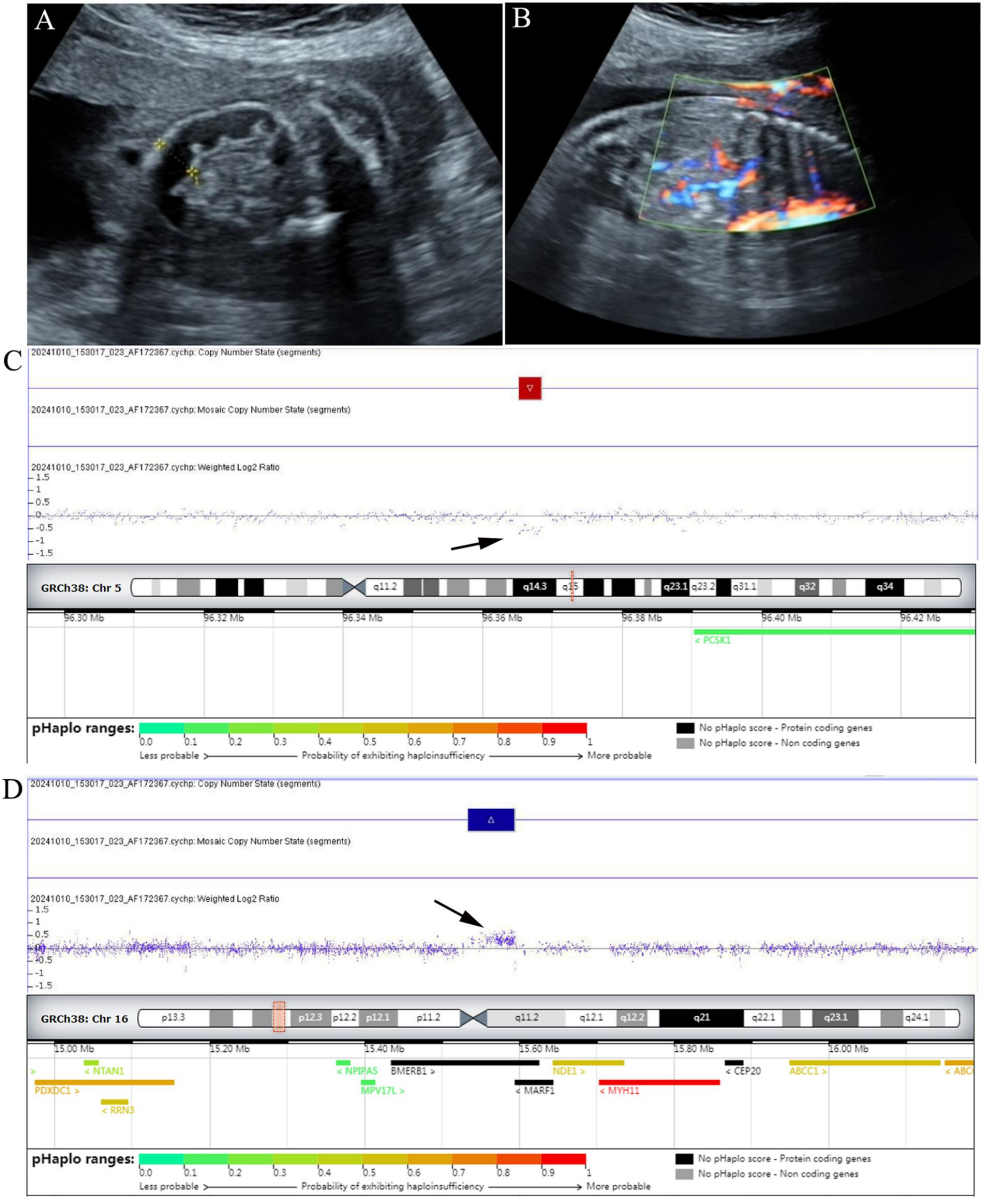
Ultrasound findings and chromosomal microarray analysis results in a fetus with 5q15 microdeletion and 16p13.11 microduplication. **(A,B)** Ultrasound examination results indicated malrotation of the intestine with midgut volvulus in Case 6. **(C)** A 136.1 Kb microdeletion in 5q15 region [arr[GRCh37]5q15(95,630,432_95,766,573) × 1] was identified in the fetus using CMA. **(D)** CMA results also revealed a 1.2 Mb microduplication [arr[GRCh37]16p13.11(15,058,821_16,282,869) × 3] in the fetus.

CMA confirmed all chromosomal aneuploies detected by karyotype analysis. As shown in Table 1, CMA identified 6 additional pCNVs/lpCNVs that were missed by karyotype analysis (17q12 microdeletion/microduplication, 16p11.2 microduplication, 22q11.21q11.22 microdeletion, 2q13 microdeletion, and 16p13.11 microduplication), resulting in an incremental diagnostic yield of 7.89% (6/76) for CMA (*P* = 0.031). Notably, Case 6 harbored a 136.1 Kb microdeletion in the 5q15 region [arr[GRCh37]5q15(95,630,432_95,766,573) × 1] and a 1.2 Mb microduplication in the 16p13.11 region [arr[GRCh37]16p13.11(15,058,821_16,282,869) × 3] (Figure 1). The 5q15 microdeletion was classified as VUS and inherited from the mother, while the 16p13.11 microduplication was classified as pCNVs and inherited from the father. Parental CMA verification was performed for an additional 3 pCNVs/lpCNVs cases, revealing 1 *de novo* variant, 1 paternal inheritance, and 1 maternal inheritance, all parents exhibited normal clinical phenotypes.

**Table 1:** Pathogenic/likely pathogenic CNVs identified in the fetuses with digestive system malformations.

Cases	CMA results	Size	Covering region	Origin	Classification	Prenatal ultrasound examination results	Pregnancy outcome
Case 1	arr[GRCh37]22q11.21q11.22 (21,804,596_22,962,962) × 1	1.1 Mb	22q11.2 recurrent region (distal type I, D-E or D-F)	*De novo*	LP	Duodenal atresia, femur and humerus shorter than the corresponding gestational age	TOP
Case 2	arr[GRCh37]17q12 (34,822,466_36,243,365) × 3	1.4 Mb	17q12 recurrent (RCAD syndrome) region (includes *HNF1B*)	Maternal	P	Duodenal obstruction	Stillbirth
Case 3	arr[GRCh37]2q13 (111,371,702_113,142,794) × 1	1.7 Mb	2q13 recurrent region (distal) (includes *BCL2L11*)	/	LP	Cystic echo in the hepatic hilum, choledochal cyst	Loss to follow up
Case 4	arr[GRCh37]16p11.2 (29,581,102_30,190,029) × 3	608.9 Kb	16p11.2 recurrent region (proximal, BP4-BP5) (includes *TBX6*)	Paternal	P	Intestinal duplication, double gallbladder, lateral ventricular dilatation	Born
Case 5	arr[GRCh37]17q12 (34,822,466_36,418,529) × 1	1.5 Mb	17q12 recurrent (RCAD syndrome) region (includes *HNF1B*)	/	P	Duodenal obstruction	TOP
Case 6	arr[GRCh37]5q15 (95,630,432_95,766,573) × 1 arr[GRCh37]16p13.11 (15,058,821_16,282,869) × 3	136.1 Kb 1.2 Mb	/ 16p13.11 recurrent region (BP1-BP3, BP2-BP3, or BP2-BP4) (includes *MYH11*)	Maternal Paternal	VUS LP	Intestinal malrotation with midgut volvulus	TOP

P, pathogenic; LP, likely pathogenic; VUS, variants of uncertain significance; TOP, termination of pregnancy; CMA, chromosomal microarray analysis.

As listed in Table 2, seven cases with VUS were also detected. Among them, three cases underwent parental CMA analysis, and all of them inherited the CNVs from their clinically normal parents, two of which were paternal, and the other one inherited from the mother.

**Table 2:** Variants of uncertain significance detected in the enrolled fetuses with digestive system malformations (DSMs).

Cases	CMA results	Size	Origin	Pathogenicity	Prenatal ultrasound examination results	Pregnancy outcome
Case 7	arr[GRCh37]22q13.2 (42,379,812_42,471,979) × 1	92.1 Kb	Maternal	VUS	Intestinal duplication, tricuspid regurgitation, increased echogenicity in the heart	Born
Case 8	arr[GRCh37]2p16.3 (51,047,025_51,352,282) × 1	305.2 Kb	/	VUS	Duodenal obstruction, increased echogenicity in the heart	TOP
Case 9	arr[GRCh37]18p11.21 (10,996,172_11,497,721) × 3	501.5 Kb	/	VUS	Ventriculomegaly, intestinal echogenicity, DSMs	Loss to follow up
Case 10	arr[GRCh37]2q21.1 (131,461,909_132,028,904) × 3	566.9 Kb	/	VUS	Intestinal duplication	TOP
Case 11	arr[GRCh37]6p25.1 (5,335,472_5,443,538) × 1	108.0 Kb	/	VUS	Duodenal obstruction, small cavum septi pellucidi	Born
Case 12	arr[GRCh37]12q24.13q24.21 (113,652,776_114,683,061) × 1	1.0 Mb	Paternal	VUS	Choledochal cyst	Loss to follow up
Case 13	arr[GRCh37]16q23.1 (75,217,775_75,589,018) × 1	371.2 Kb	Paternal	VUS	Omphalocele, increased echogenicity in the heart	Born

VUS, variants of uncertain significance; TOP, termination of pregnancy; CMA, chromosomal microarray analysis.

### Chromosomal aberration rates in the subgroups

In this study, a chromosomal aberration detection rate of 14.47% (11/76) was observed. As presented in Table 3, the chromosomal aberration detection rates in three subgroups were also analyzed. In the DSMs combined with other structural anomalies subgroup, none of pCNVs/lpCNVs was detected, with one case harboring T-21 detected, reaching a chromosomal aberration detection rate of 11.11% (1/9). Although the pCNVs/lpCNVs detection rate was higher in the isolated DSMs group (Table 1), no significant difference was observed among the subgroups (12.12% vs. 5.88% vs. 0.00%, *P* = 0.483). In addition, there was also no significant difference in total chromosomal aberration detection rates among the subgroups (15.15% vs. 14.71% vs. 11.11%, *P* = 1.000). Notably, when stratified by anatomical location, the abdominal wall anomalies group demonstrated a higher detection rate of karyotype abnormalities (2/8, 25.00%), whereas the gastric and duodenal anomalies and small intestinal anomalies groups exhibited higher detection rates of pCNVs/lpCNVs (Table 3).

**Table 3:** Karyotype abnormalities and copy number variants detected in the fetuses with digestive system malformations (DSMs).

DSMs	Cases	T21	T18	Balanced translocation	pCNVs/lpCNVs	Karyotype abnormalities detection rate	pCNVs/ lpCNVs detection rate	Chromosomal aberration detection rate
Group based on associated anomalies								
isolated	33	1	0	0	4	3.03%	12.12%	15.15%
combined with soft marker anomalies	34	1	1	1	2	8.82%	5.88%	14.71%
combined with other structural anomalies	9	1	0	0	0	11.11%	0.00%	11.11%
Group based on anatomical location								
gastric and duodenal anomalies	27	2	0	0	3	7.41%	11.11%	18.52%
small intestinal anomalies	19	0	0	1	2	5.26%	10.53%	15.79%
hepatobiliary anomalies	15	0	0	0	1	0.00%	6.67%	6.67%
abdominal wall anomalies	8	1	1	0	0	25.00%	0.00%	25.00%
other subgroups	7	0	0	0	0	0.00%	0.00%	0.00%
Total	76	3	1	1	6	6.58%	7.89%	14.47%

### Follow-Up of pregnancy outcomes and postnatal results

Follow-up data were successfully obtained for 57 cases. All fetuses with chromosomal aneuploies underwent pregnancy termination. Among the pCNVs/lpCNVs cases, 3 opted for termination, while the remaining continued their pregnancies (Table 1): 1 case resulted in stillbirth, 1 was lost to follow-up, and 1 infant was born with DSMs requiring surgery, but developmental delay was observed at the 10-month follow-up (Case 4). Of the 7 cases of VUS, 2 chose termination, 2 were lost to follow-up, and 3 continued pregnancies: 1 infant was born with DSMs requiring surgery (Case 7), and 2 exhibited normal clinical phenotypes. Among the 59 cases with normal CMA results, 43 were successfully followed up: 14 opted for termination, and 29 continued pregnancies. However, 1 fetus died unexpectedly during childbirth, 1 was born preterm with pulmonary hypoplasia, and 1 had a congenital heart defect. Additionally, 11 infants underwent surgery for DSMs postnatally: 10 exhibited well postoperative recovery and normal development, while 1 died at 1 year of age after two surgeries. The remaining 15 cases did not require surgical intervention and currently exhibit normal clinical features and developmental milestones.

## Discussion

Digestive system malformations are a major component of fetal congenital anomalies and are clinically prevalent. Previous studies have established a correlation between DSMs and chromosomal abnormalities, with relatively high detection rates (9). In the present study, an incremental diagnostic yield of 7.89% (6/76) for CMA over karyotyping was observed (*P* = 0.031). The overall chromosomal aberration detection rate was 14.47%, which was consistent with the prior reports (11.9%) (9).

In this study, four cases of chromosomal aneuploies were identified, among them, T-21 was the most common chromosomal abnormality, which was consistent with a previous report (2), but inconsistent with the other study (9). Previous studies have indicated the associations between esophageal atresia and T-18 syndrome, as well as between duodenal obstruction and T-21 syndrome (5). Similarly, in this study, duodenal obstruction was also observed in one fetus with T-21 syndrome. A previous study indicated that chromosomal anomalies may significantly increase in the DSMs combined with other system abnormalities group (9). Notably, in the present study, one case of T21 was accompanied by abnormalities of other systems, while the remaining three cases with chromosomal aneuploies presented as isolated DSMs, which may provide additional information for prenatal counseling.

Presently, extremely limited reports are available in the literature for the etiology diagnosis of fetal DSMs using CMA. A previous study conducted by Liang et al. (9) enrolled 126 fetuses with DSMs for prenatal genetic testing and revealed an additional 4 cases of pCNVs over karyotype analysis. The other study (2) enrolled a large cohort of 517 DSMs fetuses and identified an additional 11 cases with pCNVs, reaching a total detection rate of 2.12% (11/517). In this study, a higher pCNVs detection rate was observed, with 6 additional pCNVs/lpCNVs (17q12 microdeletion/microduplication, 16p11.2 microduplication, 22q11.21q11.22 microdeletion, 2q13 microdeletion and 16p13.11 microduplication) identified, reaching a 7.89% (6/76) incremental yield diagnosis of CMA over karyotype analysis.

Notably, the present study identified two fetuses with 17q12 CNVs, which were enriched in the case series, which was similar to a previous study (2). 17q12 microdeletion syndrome is a rare chromosomal anomaly syndrome typically characterized as renal cystic disease, maturity onset diabetes of the young type 5, and neurodevelopmental disorders (16), with an incomplete penetrance of 63% (17). However, the 17q12 microduplication syndrome exhibits a wide phenotypic spectrum, ranging from mild to severe phenotypes and a reduced penetrance of 20% (17, 18). Interestingly, both cases harboring 17q12 CNVs presented with isolated duodenal obstruction during the prenatal period, which may yield valuable insights for prenatal genetic diagnosis. Furthermore, isolated digestive obstruction or duodenal atresia has also been documented in fetuses with 17q12 microduplication in previous studies (2, 19). In addition, other DSMs, including anorectal malformations and choledochal cysts, have been reported to be associated with 17q12 CNVs (20, 21). In the present study, Case 4 with 16p11.2 microduplication was postnatally diagnosed with intestinal duplication, double gallbladder, and developmental delay. The developmental delay may be due to the 16p11.2 microduplication in the patient, while, little is known about the relationship between 16p11.2 microduplication and DSMs. In addition, whole exome sequencing was conducted to detect possible sequenced variants in the patient, with none of the pathogenic sequenced variants identified (data not shown). Moreover, a previous study conducted by Sagi-Dain et al. (22) identified 16p11.2 microduplication in patient with isolated non-visualized gallbladder. Thus, the DSMs observed in the Case 4 of the present study may be attributed to 16p11.2 microduplication syndrome, but more functional evidence and additional clinical studies are warranted. Additionally, consistent with a previous report (23), 22q11.21q11.22 microdeletion syndrome was also identified in the fetus presenting with DSMs. Notably, the only prior study by Salehi et al. (24) suggested an association between 16p13.11 microduplication syndrome and intestinal malrotation. Our current findings further strengthen the potential link between 16p13.11 microduplication syndrome and intestinal malrotation. However, the relationship between 2q13 microdeletion and DSMs was unknown, and whether 2q13 microdeletion was responsible for DSMs needs more investigation.

At present, there are still controversies regarding the conclusions of subgroup studies on the detection rate of chromosomal aberration in DSMs. A previous study indicated that the detection rate of chromosomal aberration in isolated DSMs subgroup is significantly lower than that in the group of DSMs combined with other abnormalities (9), while no significant difference has been found in other studies (2). A higher chromosomal aberration detection rate was observed in the isolated DSMs group but without significant difference among the groups, which may provide useful information for further genetic counseling. Furthermore, our results demonstrated that the gastric and duodenal anomalies and small intestinal anomalies groups had higher detection rates of pCNVs/lpCNVs, although validation in larger cohorts is warranted. In addition, a previous study indicated that children with DSMs may face the risk of neurodevelopmental impairment due to the impact of surgical treatment, and the neurodevelopmental outcomes are associated with the number of surgeries and total length of hospital stay (25). Therefore, in subsequent follow-ups, adequate attention should be paid to the pediatric patients with DSMs, and the relevant risks should also be fully informed during prenatal clinical consultations.

In the present study, seven cases with VUS were detected, reaching a 9.21% detection rate, which was similar to another study (2). Similar to the previous studies, most of the VUS detected in the present study are hereditary (26, 27). The clinical phenotype associated with fetal VUS remains uncertain, and additional research is required to clarify the genotype-phenotype relationship. Despite that parental origin verification, prenatal ultrasound and postnatal follow-up would be helpful for the clinical counseling of the detected VUS. In addition, this study also has some limitations, including the limited number of DSMs cases included in the study and the relatively low successful follow-up rate.

In conclusion, a total of 76 fetuses diagnosed with DSMs were identified from 7,497 pregnant cases, and several pCNVs/lpCNVs closely link to DSMs were identified. Our findings demonstrated distinct disease correlations, including 16p11.2 microduplication syndrome and DSMs, 17q12 microdeletion/microduplication and duodenal obstruction, and 16p13.11 microduplication syndrome and intestinal malrotation. Nevertheless, further functional evidence is needed to validate these genetic associations. This study offers additional genetic insights into fetuses with DSMs and deepens our understanding of genotype-phenotype correlations in this clinical entity.

## Data Availability

The raw data supporting the conclusions of this article will be made available by the authors, without undue reservation.
